# Potential prognostic value of biomarkers in lavage, sputum and serum in a five year clinical follow-up of smokers with and without COPD

**DOI:** 10.1186/1471-2466-14-30

**Published:** 2014-03-01

**Authors:** Olaf Holz, Benjamin Waschki, Stefan Roepcke, Henrik Watz, Gereon Lauer, Cornelia Faulenbach, Jens M Hohlfeld

**Affiliations:** 1 Department of Clinical Airway Research, Fraunhofer Institute of Toxicology and Experimental Medicine, Hannover 30625, Germany; 2 Biomedical Research in Endstage and Obstructive Lung Disease Hannover (BREATH), Member of the German Center for Lung Research, Hannover, Germany; 3 Pulmonary Research Institute at LungenClinic Grosshansdorf, LungenClinic Grosshansdorf, Airway Research Centre North (ARCN), Member of the German Center for Lung Research, Grosshansdorf, Germany; 4 Takeda Pharmaceuticals International GmbH, Zürich, Switzerland

**Keywords:** Airway inflammation, Clinical value, Lung function

## Abstract

**Background:**

The aim of this study was to test whether repeatable biomarkers collected from serum, bronchoalveolar lavage (BAL) and sputum of healthy smokers and smokers with COPD would have a prognostic value with respect to the decline in lung function over a 5 year period.

**Methods:**

In 2006/2007 we had repeatedly collected serum, BAL and sputum of 23 healthy smokers and 24 smokers with COPD (GOLD II) and analysed a panel of more than 100 different parameters. In 2012 we reinvited these subjects to assess the change in lung function to enable the investigation of the potential prognostic value of the 2006/2007 markers and to determine the long-term repeatability of selected blood and serum markers. In this follow-up study we performed body-plethysmography, a blood gas analysis and collected blood and urine samples. The change in lung function was compared with 67 markers from BAL, sputum, serum and whole blood that were shown in the 2006/2007 assessment to be repeatable over a 6 week period.

**Results:**

We were able to recruit 13 (54%) smokers with COPD and 11 (48%) former healthy smokers that participated in the 2006/2007 study. The decline in lung function was larger in COPD smokers; five of them changed to GOLD III, one to GOLD IV. Two healthy smokers changed to GOLD I. Blood cells, serum von Willebrand factor and alpha-1-antitrypsin showed a good repeatability over 5 years. In COPD smokers a weak correlation between 2006/2007 sputum markers of neutrophilic inflammation and the 5 year change in FEV_1_/FVC was found.

**Conclusions:**

Our data suggests that inter-individual and group differences are maintained over a five year period. Despite the large panel of markers available for this analysis, a potential prognostic value appears to exist only for some sputum inflammatory markers. If these data can be confirmed in larger COPD cohorts, it would emphasize the value of sputum markers in clinical trials and support the assumption that an anti-inflammatory treatment can have long term benefits in COPD.

## Background

In 2006/2007 we performed a large biomarker study, in which we included two well matched groups of smokers, one group with COPD (GOLD II) and one group without [1]. Samples from all relevant compartments (sputum, bronchoalveolar lavage (BAL), mucosal biopsies, serum, whole blood, and urine) were collected twice within a period of 6 weeks to assess the repeatability of the large panel of markers. It was the aim to find robust markers or combinations of markers which reflect the underlying pathological processes in COPD and could therefore be used as potential novel targets for treatment and as markers in clinical trials with novel anti-inflammatory compounds for COPD. In addition, we wanted to know to what extent serum markers relate to inflammatory markers within the airways to find more easily accessible biomarkers for clinical trials.

The design of the 2006/2007 study was not suited to provide information about the prognostic value of the markers with respect to the long-term functional outcome of patients with COPD. Markers with the potential to serve as surrogate markers for lung function are needed to enable shorter and therefore safer clinical trials especially for novel anti-inflammatory compounds.

There is data available for serum markers for e.g. C-reactive protein (CRP), fibrinogen and adiponectin with respect to their predictive value on the decline of lung function, exacerbation rate and mortality [2-6]. To our knowledge, no prospective study exists with respect to the predictive value of markers assessed in BAL and sputum, except for a COPD study looking at predictors in sputum for exacerbations induced by steroid withdrawal [7]. As such comprehensive panels in different compartments are generally not possible to be tested in larger cohort studies; we considered it worth to address this question despite the comparatively low number of subjects available. In addition, we aimed to determine the long-term repeatability of a number of blood and serum biomarkers in this five year follow-up trial.

The data obtained from this re-evaluation could provide valuable information for large on-going or past COPD trials like ECLIPSE [8] or SPIROMICS [9] for which sputum data is available and were these preliminary findings could be validated.

## Methods

### Subjects

We invited all of the 47 participants of our initial biomarker study [1]. Thirteen (54%) smokers with COPD and 11 (48%) former healthy smokers were recruited for this follow-up study; the remaining subjects could not be reached despite intensive recruitment efforts by phone and mailing. Only one of the contacted subjects declined to participate in the follow-up. The study was conducted in accordance with Good Clinical Practice and the Declaration of Helsinki. Subjects gave their written informed consent. The study was approved by the Ethical Committee of Hannover Medical School. The same applies for the study conducted in Grosshansdorf, which was approved by the Ethical Committee of the Medical Chamber of Schleswig-Holstein. Data from a subgroup of this cohort was used for comparison.

### Study design

After providing informed consent, subjects underwent a thorough physical examination and history assessment of the past 5 years using a non-validated 10 question questionnaire with analogue scales for information about changes of different aspects in disease activity. Vital signs, safety laboratory tests, blood gas analysis, 12-lead-electrocardiogram, and body-plethysmography were also performed. A blood sample was taken for the analysis of selected serum markers and the current smoking status was controlled by a urine cotinine measurement. The low number of subjects available for the re-evaluation in 2012 did not ethically justify a repetition of the more invasive procedures, but allowed to derive data for the long term repeatability of blood and selected serum markers.

### Questionnaire

Subjects rated their estimation on a fixed scale from 1 (no) to 10 (yes). The following questions were asked (abbreviated): Did you experience (1) more cough over the past 5 years? (2) more sputum? (3) a change in sputum color? (4) more respiratory infections? (5) more days with bad lung function? (6) Did your general health, or (7) your exercise/activity capacity deteriorate over the last 5 years? (8) Did your overall quality of life change over the past 5 years?

### Biomarker analysis

The analysis of leukotriene B4 (LTB4), Interleukin 6 (IL6), alpha-1-antitrypsin (A1AT), insulin growth factor 2 (IGF2) and von Willebrand Factor (VWF) was performed by ELISA. We measured IGF2 and VWF in stored serum samples of a well characterized independent COPD cohort [10] to test if we could reproduce our findings. In addition, we tested leukocyte and hematology parameters in this cohort as well as the available serum/plasma markers (fibrinogen, CRP, leptin, adeponectin, IL6) that were analyzed in 2006 for their relationship to the lung function decline over three years.

### Statistical analysis

To assess the repeatability of the markers that were analysed both in 2006/2007 and in 2012 the correlation coefficient and the intra-class correlation coefficient was computed. The intra-class correlation coefficients (ICC) were derived from one-way ANOVA tables as the ratio of variance among subjects to total variance based on 2 measurements over the 5 year period ([11]: (BMS-WMS/2)/((BMS-WMS/2) + WMS)); BMS = between group mean square, WMS = within group mean square).

The change in lung function was taken as the major clinical outcome variable. Due to the fact that 2006/2007 post-bronchodilator data was only available for the COPD patients, we calculated the absolute change in pre-bronchodilator FEV_1_ (L), the absolute change in FEV_1_ (%pred.) and the absolute change in FEV_1_/FVC (%). We consider this to be a valid approach, as for COPD patients the pre- and post-bronchodilator changes in FEV_1_ (L), FEV_1_ (%pred.) and FEV_1_/FVC were significantly correlated (all r ≥ 0.92, ICC ≥ 0.92, p < 0.001). We correlated the changes in lung function only with markers, which had acceptable repeatability in the 2 visits 2006/2007 (10 BAL-, 10 induced sputum -, 24 serum markers listed in Additional file 1: Table S2, plus 23 blood parameters, for which the repeatability was not assessed). The analysis was performed for all subjects and for both groups separately.

## Results

### Subjects

Thirteen (54%) smokers with COPD and 11 (48%) former healthy smokers could be recruited for this follow-up study. Table 1 gives the 2006/2007 demographics for all subjects and for the re-evaluated subgroup both for 2006/2007 and 2012. Additional file 1: Table S1 provides information on medication, and comorbidities as derived from the medical history. The median (interquartile range, IQR) time between visits was 5.0 (4.7, 5.2) years.

**Table 1:** Demographics

	**2006/2007 Complete cohort**		**2006/2007 Data for subjects studied in 2012**		**2012 Data**	
	**Healthy smokers (N = 23)**	**COPD smokers (N = 24)**	**Healthy smokers (N = 11)**	**COPD smokers (N = 13)**	**Healthy smokers (N = 11)**	**COPD smokers (N = 13)**
Female/male	6/17	6/18	4/7	4/9	4/7	4/9
Age [years]^a^	54 (42, 65)	54 (46, 68)	56 (51, 61)	53 (50, 56)	60 (56, 66)	58 (55, 61)
BMI [kg/m^2^]	25.4 ± 2.5	25.3 ± 3.4	25.5 ± 1.8	24.4 ± 3.0	26.7 ± 3.0	25.5 ± 3.9
Pack-years &	34.0 (21.0)	48.0 (15.5)***	32.0 (21.0)	47.0 (15.0)	37.5 (16.8)	50.1 (16.7)
Cotinine (ng/mL)	1262 ± 722	1561 ± 968	1232 ± 548	1620 ± 748	867 ± 659	1585 ± 678
FEV_1_ [L]	3.8 ± 0.8	2.0 ± 0.3***	3.5 ± 0.6	2.0 ± 0.3	3.2 ± 0.5	1.5 ± 0.5
FEV_1_ % pred.	112.5 ± 14.1	60.5 ± 6.8***	114.9 ± 12.8	60.7 ± 5.5	109,6 ± 14.2	47.3 ± 15.0
FVC [L]	5.1 ± 1.0	4.2 ± 0.9**	4.7 ± 0.9	4.3 ± 0.8	4.5 ± 0.7	3.6 ± 0.8
FEV_1_/FVC [%]	75.4 ± 5.0	48.7 ± 7.4***	74.3 ± 5.1	48.2 ± 6.7	71.1 ± 4.2	41.0 ±11.7
pO2 [mm Hg]	82.9 ± 9.7	73.3 ± 5.3***	81.2 ± 9.2	75.1 ± 5.7	76.5 ± 7.4	69,2 ± 7.6

In 2012: COPD smokers: 2 quit smoking, 2 reduced smoking; healthy smokers: 2 quit smoking, 1 reduced smoking.

^a^:presented as mean (minimum, maximum), all other data is presented as mean ± SD, ** = p < 0.01, *** = p < 0.005 & median (IQR).

### Changes in lung function over a 5 year period

With respect to the demographic data (Table 1), neither the healthy smokers nor the smokers with COPD that could be recruited for this follow-up investigation, differed from the groups studied in 2006/2007. In both groups two subjects stopped smoking, but a significant correlation for urine cotinine between 2006/2007 and 2012 indicates that no major changes in smoking behavior occurred (r = 0.79, p < 0.001).

**Figure 1:**
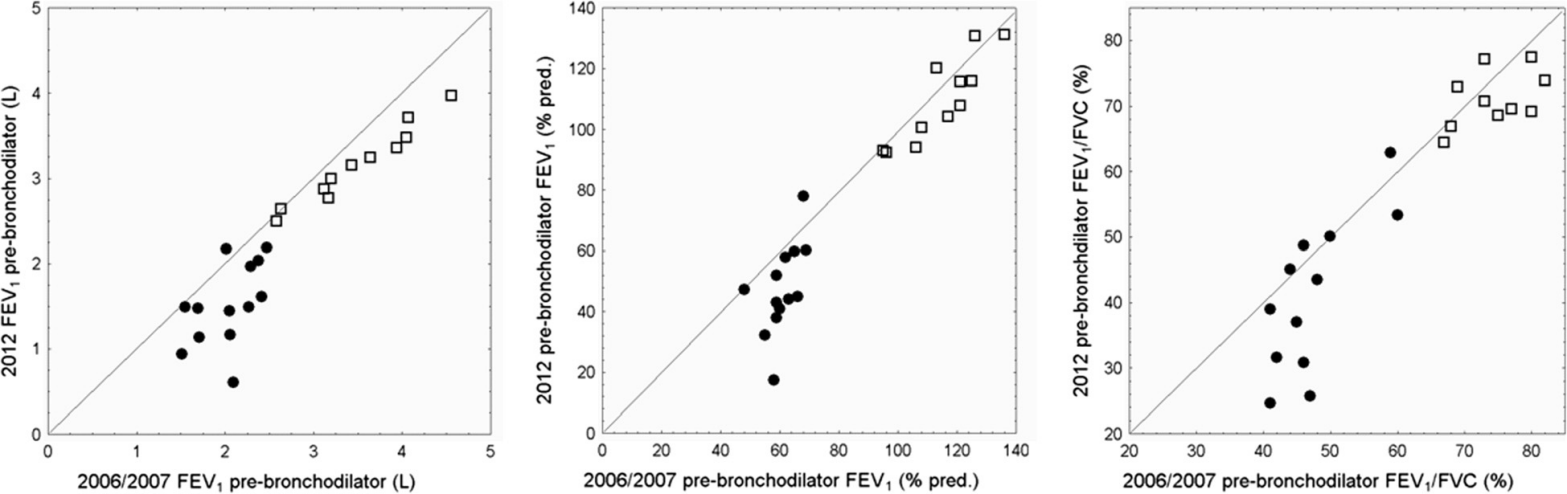
**Change in pre-bronchodilator FEV**_**1 **_**(L), FEV**_**1 **_**(% pred.) and FEV**_**1**_**/FVC (left, middle, right) from 2006/2007 to 2012 in smokers with COPD (circles) and former healthy smokers (open squares).** A larger drop below the line of identity indicates a larger decline in the respective lung function level. The changes with respect to GOLD category as listed in the text were done using post-bronchodilator values, which are slightly higher as compared to the pre-bronchodilator values displayed in this figure.

Both groups showed a significant decline in lung function (pre-bronchodilator) over the five year period, which was more pronounced in smokers with COPD (Figure 1). The median (IQR) change in pre-bronchodilator FEV_1_ was −0.57 (0.49) L and −0.35 (0.37) L in smokers with and without COPD, respectively (both p < 0.005, Wilcoxon test). The absolute decline in pre-bronchodilator FEV_1_ %pred. was −16.1 (15.8) and −5.4 (10.0) % (p < 0.01, p < 0.05), and the absolute decline in pre-bronchodilator FEV_1_/FVC was −5.6 (13.4) and −2.6 (6.3) % (p < 0.05, p = 0.09), respectively. Five smokers with COPD changed to GOLD III, one to GOLD IV. Two former healthy smokers changed to GOLD I (FEV_1_/FVC post-bronchodilator < 70%). The results of the questionnaire were compatible with this observation, showing significantly higher levels on the analog scales for questions related to general health, cough, exercise tolerance, general lung health, and quality of life.

### Repeatability of whole blood parameters over a 5 year period

**Figure 2:**
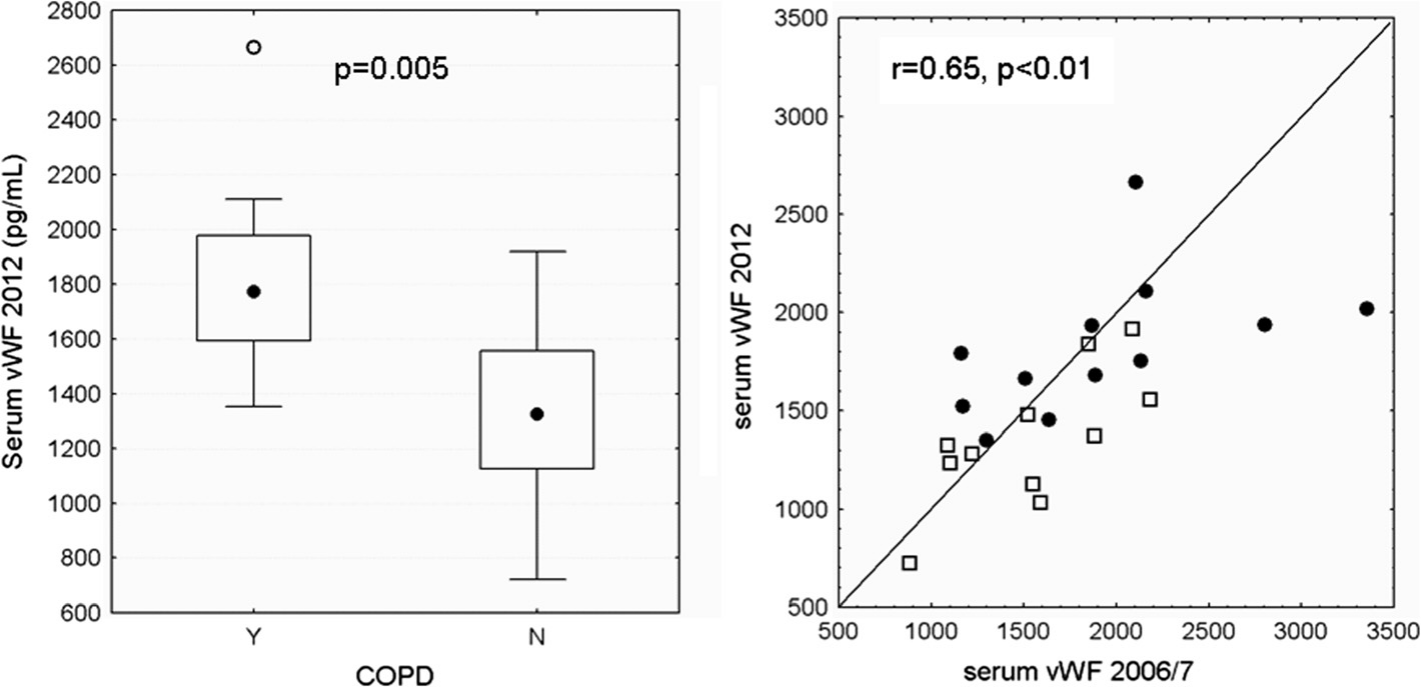
**A significant difference between smokers with and without COPD for the level of serum VWF was shown in 2006/2007 and this difference was maintained in 2012 (left).** The correlation between 2006/2007 and 2012 levels of VWF was significant (right). The data is shown for smokers with (circles) and without (open squares) COPD and with the line of identity.

Among the cellular blood parameters that were analyzed at both time points (Table 2), there was only a slight decrease in the percentage of blood monocytes in former healthy smokers (p < 0.05) and a small increase of mean corpuscular hemoglobin (MCH) levels in both groups, with more pronounced increases in smokers with COPD (p < 0.005). Table 2 gives the correlation and the ICCs for markers that were analyzed both in 2006/2007 and 2012. Additional file 2: Figure S1 shows the correlation between time points for blood leukocytes and the percentage of blood monocytes. In line with the 2006/2007 data, we found significantly higher serum levels for A1AT, IL6 and VWF in smokers with COPD, while the observed difference between groups for LTB4 could not be reproduced. Levels were remarkably stable for serum VWF (Figure 2). Interestingly, the 2 healthy smokers that progressed to COPD GOLD 1 had the highest control group levels of A1AT and IL6 in 2012.

**Table 2:** Repeatability of hematology data and selected serum proteins

	**Parameter**	**Unit**	**r**	**ICC**	**Healthy smokers**	**COPD smokers**	**Healthy vs. COPD**		**2006/2007 vs. 2012**^**$**^		
							**2006/2007**^**#**^	**2012&**	**All**	**Healthy**	**COPD**
										**smokers**	**smokers**
Whole blood	Leukocytes	10^9^/L	0.84	0.85	7.1 (4.8-7.9)	7.4 (6.8-9.3)					
	Neutrophils	(%)	0.54	0.50	54.3 (51.4-63.8)	62.6 (58.4-64.4)					
	Monocytes	(%)	0.72	0.70	8.4 (8.2-9.0)	7.7 (6.9-9.4)				p = 0.03	
	Erythrocytes	10^12^/L	0.79	0.78	4.8 (4.6-5.0)	4.8 (4.5-5.0)					
	Thrombocytes	10^9^/L	0.86	0.86	248.0 (208.0-272.0)	208.0 (176.0-257.0)					
	Anisocytosis		0.81	0.82	45.0 (43.0-47.0)	46.0 (46.0-47.0)	p = 0.014				
	Hemoglobin	g/dL	0.80	0.81	14.8 (14.0-15.1)	15.0 (14.2-15.7)					
	Hematocrit	%	0.73	0.73	44.0 (42.0-44.0)	44.0 (43.0-47.0)					
	MCH	pg	0.83	0.68	30.8 (29.2-31.9)	31.5 (30.7-32.6)			p < 0.001	p = 0.03	p < 0.005
	MCV	10^9^ μL	0.91	0.91	89.1 (87.0-94.8)	93.0 (91.1-94.4)	p = 0.008				
Serum	Serum creatinine	mg/dL	0.88	0.71	0.9 (0.8-1.0)	0.8 (0.7-0.9)			p = 0.006	p = 0.003	
	ALP	U/L	0.86	0.78	79.0 (65.0-95.0)	89.0 (70.5-97.5)					
	AST/GOT	U/L	0.68	0.79	25.0 (24.0-30.0)	23.5 (20.5-29.5)					
	ALT/GPT	U/L	0.67	0.58	23.0 (21.0-38.0)	21.5 (18.5-33.5)					
	G-GT	U/L	0.87	0.82	27.0 (20.0-30.0)	42.5 (22.0-56.0)	p = 0.09	p = 0.05	p = 0.01		p = 0.03
	LTB4	μg/mL	0.01	−0.02	1.5 (0.9-2.1)	1.6 (1.2-2.0)	m: p = 0.0051		nd	nd	nd
	A1AT	ng/mL	0.74	−0.53	767.9 (707.5-844.5)	870.9 (828.8-906.6)	m: p = 0.014	p = 0.02	nd	nd	nd
	IL6	pg/mL	0.25	−0.34	1.6 (1.1-3.4)	3.2 (2.0-4.0)	p = 0.002	p = 0.02	nd	nd	nd
	VWF	U/mL	0.69	0.67	1.3 (1.1-1.6)	1.8 (1.6-2.0)	p = 0.003	p = 0.004			

MCH: mean corpuscular/cellular hemoglobin. MCV: mean corpuscular/cell volume, ALT/GPT:Alanin-Aminotransferase/Glutamat-Pyruvat-Transaminase.

AST/GOT: Aspartat-Aminotransferase/Glutamat-Oxalacetat-Transaminase, ALP: Alkaline phosphatase, GGT: Gamma-glutamyltransferase.

#Roepcke et al. PLOS One 2012 [1] Table 4/Table S8. m = male subjects, & Mann–Whitney-U-test, $ Wilcoxon-test.

r = correlation coeficient. ICC = intraclass correlation coeficient comparing 2006/2007 and 2012 data of all subjects.

nd = not done. due to deviations based on differences in ELISA batches.

### Relationship between markers analyzed in 2006/2007 and the 5 year decline in lung function

Additional file 1: Table S2 lists all those parameters with acceptable repeatability in 2006/2007 (ICC > 0.6) in smokers with and without COPD. For a full list of markers that were assessed in 2006/2007 refer to Roepcke et al. [1]. For all markers listed in Table 3 as well as for whole blood markers and hematology data we analyzed the relationship to the decline in pre-bronchodilator FEV_1_ (L), FEV_1_ (%pred.) and the change in the FEV_1_/FVC ratio. Table 3 provides a list for all markers, which showed a significant correlation coefficient >0.50. All these correlations were checked visually to control for a bias due to outliers.

**Table 3:** Correlation between markers assessed 2006/2007 and the decline in lung function

	**FEV**_**1 **_**(L)**			**FEV**_**1 **_**(%pred.)**			**FEV**_**1**_**/FVC (%)**		
	**All**	**COPD smokers**	**Healthy smokers**	**All**	**COPD smokers**	**Healthy smokers**	**All**	**COPD smokers**	**Healthy smokers**
**BAL:**									
Calprotectin							−0.53**		
IL8									−0.72*
**IS:**									
MMP7		−0.6		−0.53*	−0.62		−0.58**	−0.73**	
MMP9								−0.66*	
MMP9/TP								−0.65*	
A1AT			−0.72			−0.75			
HSA			−0.84**			−0.88**			
IL-6						−0.76			
**Serum:**									
IGF2		0.74***			0.72**			0.78***	
Leptin		0.72**			0.7**			0.75***	
Creatinine			−0.65						
**Blood:**									
ALP					0.65*				
HCT			−0.77**			−0.82***			
HGB			−0.78***			−0.80***			
RBC			−0.8***			−0.79***			
Monocytes									−0.74*

Only significant correlations are displayed and are either p < 0.05, or as marked: *p < 0.02, **p ≤ 0.01, ***p ≤ 0.005.

BAL = bronchial alveolar lavage, IS = induced sputum, Calprot. = Calprotectin, HSA = human serum albumin, HCT = hematocrit, HGB = hemoglobin, MMP = matrix metalloproteinase, RBC = red blood cells, ALP: Alkaline phosphatase. A negative correlation means that a stronger decline in lung function over the 5 year period is associated with higher levels of the respective parameter (e.g. sputum MMP7). A positive correlation indicates that lower levels of the respective parameter are associated with a stronger decline in lung function (e.g. IGF2).

In 2006/2007 we also computed cumulative scores based on different BAL or sputum markers [1]. No relationship was detected between lung function changes and the computed BAL score. For the sputum cumulative score (based on the levels of A1AT, IL6, MMP7, HSA and sputum neutrophils), which showed a better repeatability over 6 weeks as compared to sputum neutrophils alone [1], we found a correlation with the decline in FEV_1_ (L) and FEV_1_ (% pred) (r = −0.78, p = 0.02; r = −0.80, p = 0.02) in former healthy smokers. However, only 8 subjects were available for this comparison.

Higher levels of BAL or induced sputum markers associated with neutrophilic airway inflammation (e.g. IL8, calprotection or matrix metalloproteases, MMPs) in 2006/2007 were associated with a larger decline in lung function over a 5 years period. For serum leptin and IGF2 a reverse relationship was found, showing that a stronger decline in lung function was related to lower levels in serum. Due to the fact that these observations were limited to subgroups and are based on small subject numbers the data needs to be interpreted with caution.

### Testing results in an independent COPD cohort

Due to the finding that serum IGF2 might be playing a protective role with respect to the decline in lung function we aimed to test this preliminary result in an independent well characterized group of smoking COPD GOLD 2 patients [10], for which 3 year follow-up data was available (Grosshansdorf cohort). Demographic data of this cohort is presented in Additional file 1: Table S3. The Grosshansdorf cohort does not include healthy smokers, therefore no re-evaluation of differences between groups could be performed. The 24 patients were on average about 10 years older, had a higher BMI (~3), and more pack-years. The median (IQR) change in FEV_1_ was −0.18 (0.24) L (p < 0.001, Wilcoxon Test). The decline in FEV_1_ (% pred.) was −3.5 (8.4) % (p < 0.0065) and the change in FEV_1_/FVC was 1.1 (8.3) %. Four of the GOLD 2 smokers progressed to GOLD 3 over the three year period. There was a comparable good repeatability for the hematology data over three years (Additional file 1: Table S4).

In these 24 COPD GOLD 2 patients, we measured IGF2 and VWF in stored serum samples, but we could not reproduce the findings in our GOLD 2 patient group. In addition, we did not find a relationship to the lung function decline over three years (exceeding r =0.50) with respect to leukocyte or hematology parameters or available serum/plasma markers (fibrinogen, CRP, leptin, adeponectin, IL6) that were analyzed in this cohort in 2006.

## Discussion

Despite the available large panel of markers from BAL, sputum, serum and blood, evidence for a potential prognostic value was found only for some sputum inflammatory markers. Naturally only low numbers of subjects are available in studies involving invasive procedures like our initial trial in 2006/2007. Therefore, the association of these markers with the decline in lung function has to be interpreted with caution. This is also reflected by the fact, that our interesting observation, that COPD patients with higher serum levels of leptin and IGF2 showed a smaller decline in lung function could not be reproduced in an independent group of COPD patients. For most hematology parameters, however, inter-individual and group differences were shown to be stable over a five year period. This was also true for the serum concentration of alpha-1-antitrypsin and von Willebrand-Factor.

The lung function decline was larger in smoking COPD patients than in former healthy smokers. The annual decline in these smokers exceeded the average amount reported in the UPLIFT study, the Hokkaido COPD cohort or the study by Higashimoto et al. [2,12,13]. These cohorts, however, only included 20-30% active smokers. The annual decline in lung function of the GOLD 2 COPD smokers from the Grosshansdorf cohort was comparable [10] and larger declines in FEV_1_ were also reported for active smokers with COPD in the ECLIPSE cohort [14]. Although the rate of individual annual decline in FEV_1_ was based on only two measurements several years apart, which could be considered as a limitation of our study, the overall magnitude of the decline rate was in agreement with other studies on actively smoking COPD patients.

While the level of sputum neutrophils in 2006/2007 was not related to the change in lung function parameters over the investigated 5 years period, we found several markers associated with neutrophilic airway inflammation in the lung, like sputum IL8, BAL calprotection or sputum MMPs, for which the concentrations in 2006/2007 correlated with a larger decline in lung function. Interestingly, 4 of the 5 sputum markers we choose in the initial analysis [1] to define an inflammatory phenotype to cover more aspects of inflammation than neutrophils alone, were among the markers that showed significant correlations with the lung function decline. Also the inflammatory score itself was significant, however, only available for 8 subjects. Overall the evidence was weak and higher correlation coefficients with lung function decline were found only in subgroups. In addition, the multiple testings have to be considered and therefore this data needs to be interpreted with caution.

Blood haematology markers have moved into the focus of biomarker studies. In the SPIROMICS initiative (Subpopulations and intermediate outcome measures in COPD study) blood cell counts and haematology variables were assessed and shown to be related to COPD severity [15]. Our data suggests that inter-individual differences of these markers in smokers with and without COPD persist over a five year period and that these markers show a good repeatability. In 2006/2007 we found differences in anisocytosis, an indicator for anaemia and the mean corpuscular volume (MCV) between healthy smokers and smoking COPD patients. In line with these findings, SPIRIOMICS reported increased levels of haemoglobin, haematocrit, MCV and leukocyte counts in COPD patients [15]. The observed relationship with lung function in healthy smokers of these haematology markers suggests that it might be worth to re-evaluate these markers in already available data of large COPD cohorts.

A good repeatability was also found for serum A1AT and VWF and the differences in serum concentrations of A1AT, VWF and IL6 between groups were still detectable, despite lower numbers of subjects available. This confirms that these markers play a role in COPD pathogenesis, but do not appear to have any prognostic value.

A positive correlation with the lung function decline was found for IGF2 and leptin, suggesting a potential protective role. For leptin this could not be observed in the independent Grosshansdorf COPD GOLD 2 cohort. As no IGF2 data was available for this group, we analysed IGF2 from stored blood samples of 2006. The levels were comparable to the levels detected in fresh samples 2012, suggesting that storage did not have a negative impact. Nevertheless, we failed to find a comparable relationship of this marker with the lung function decline.

Our negative findings with respect to the large panel of serum markers we analysed in 2006/2007 is in line with data from the much larger Hokkaido COPD cohort, where only adiponectin (of 52 plasma markers) was reported to relate to the lung function decline over 5 years [5,13]. In the ECLIPSE study a similar analysis was performed in almost 1800 patients with 7 serum markers [14]. Although significant, only a small effect for CC16 was found; CC16 being responsible for a 4 ml FEV1 decline/year. Using basically the same dataset from the ECLIPSE study Agusti et al. reported that those COPD subjects with persistently high levels of systemic inflammatory markers had a higher incidence of exacerbations and a higher rate of mortality [16]. In addition it was shown in ECLIPSE that considering serum levels of IL6 improves the predictive value of age, BODE and hospitalization history [17]. Similar data has recently been published for the follow up of COPD patients from the Copenhagen City Heart – (2 years) and General Population Study (5 years), were patients with high levels of CRP, fibrinogen and leukocyte count were shown to have a higher risk of exacerbations [18]. Serum CCL-18 levels were also found to be related to mortality in the ECLIPSE cohort [19]. There is quite a large number of plasma markers that was found to be associated with the exacerbation rate in COPD [20]. A comprehensive table which lists the evidence for 17 potential biomarkers with the respective outcomes can be found in the paper by Sin and Vestbo [21] and Koutsokera et al. [3].

## Conclusion

In summary, our study provides data about the long term repeatability of selected blood and serum markers and suggests that in current smokers with COPD there is relationship between sputum markers associated with markers of neutrophilic inflammation and lung function decline. The data could provide valuable information for large on-going or past COPD trials like ECLIPSE or SPIROMICS for which sputum data is available and where these preliminary findings could be validated.

## Competing interests

The authors declare that they have no competing interests.

## Authors’ contributions

OH, SR, GL, and JMH designed, analysed, and interpreted lab experiments. CF was the responsible physician for the recruitment and examination of the study patients. HW and BW were responsible for the Grosshansdorf COPD cohort. OH and JMH drafted the manuscript. All authors read and provided comments on draft versions of the manuscript, and approved the final manuscript version for submission.

## Pre-publication history

The pre-publication history for this paper can be accessed here:

http://www.biomedcentral.com/1471-2466/14/30/prepub

## Supplementary Material

Additional file 1: Table S1 Medication of COPD patients between 2006/2007 and 2012. **Table S2.** List of all markers, which were shown to be repeatable in 2006/2007. **Table S3.** Demographic data of an independent group of smoking COPD GOLD 2 patients from Grosshansdorf, Germany. **Table S4.** Repeatability of blood cells and hematology markers between 2006 and 2009. Click here for file

Additional file 2: Figure S1 Correlation for total blood leukocytes (left) and the percentage of monocytes (right) between 2006/2007 and 2012. Open symbols: smokers without COPD, closed symbols smokers with COPD. Click here for file
