# Supplementary material for: Potential prognostic value of biomarkers in lavage, sputum and serum in a five year clinical follow-up of smokers with and without COPD
Source: BMC Pulm Med. 2014 Mar 1;14:30. doi: 10.1186/1471-2466-14-30 (PMC4021348; doi:10.1186/1471-2466-14-30)
Supplement: Additional file 1: Table S1 — Medication of COPD patients between 2006/2007 and 2012. Table S2. List of all markers, which were shown to be repeatable in 2006/2007. Table S3. Demographic data of an independent group of smoking COPD GOLD 2 patients from Grosshansdorf, Germany. Table S4. Repeatability of blood cells and hematology markers between 2006 and 2009. [file 1471-2466-14-30-S1.docx]

**Potential prognostic value of biomarkers in lavage, sputum and serum in a five year clinical follow-up of smokers with and without COPD**

**Table S1: Medication of COPD patients between 2006/2007 and 2012**

| COPD Pat. | GOLD 2006/7 | GOLD 2012 | SABA | year | LABA | year | LAMA | year | ICS | year | PDE4-Inh. | y | Statins | year | CVD med. | year | Other | | Other comorbidities |
| --- | --- | --- | --- | --- | --- | --- | --- | --- | --- | --- | --- | --- | --- | --- | --- | --- | --- | --- | --- |
| 1 | II | III |  |  |  |  |  |  |  |  |  |  |  |  |  |  | X | | seasonal allergy |
| 2 | II | I |  |  | XX | 2011 |  |  | XX | 2011 |  |  |  |  |  |  | XX | |  |
| 3 | II | II | XX | 2005 |  |  |  |  |  |  |  |  | XX | 2010 | X ; XX | 2006/2010 | X;XX | | seas. allergy, diabetis |
| 4 | II | II | XX | 2010 |  |  |  |  |  |  |  |  |  |  |  |  | X | | depression |
| 5 | II | II | XX | 2011 | X |  | XX | 2008 | X |  |  |  |  |  |  |  | X | |  |
| 6 | II | II |  |  |  |  |  |  |  |  |  |  | XX | 2010 | X ; XX | 2010 | XX | |  |
| 7 | II | II |  |  |  |  |  |  |  |  |  |  |  |  | XX | 2011/2012 | X | | depression |
| 8 | II | III |  |  |  |  |  |  |  |  |  |  |  |  |  |  | XX | |  |
| 9 | II | II |  |  |  |  |  |  |  |  |  |  |  |  |  |  |  | |  |
| 10 | II | III | XX | 1996 |  |  | XX | 2010 |  |  |  |  |  |  | XX | 2010 |  | |  |
| 11 | II | III | XX | 2008 |  |  | XX | 2008 |  |  |  |  |  |  |  |  |  | |  |
| 12 | II | III | X |  |  |  |  |  |  |  |  |  |  |  | XX | 2007 | X | |  |
| 13 | II | IV | XX | 2008 | X |  | XX | 2009 | X |  | XX | 2011 |  |  | XX | 2008 |  | |  |
| SABA=short acting beta-agonist, LABA=long acting beta-agonist, LAMA=long-acting anti-muscarinic agents, | | | | | | | | | | | | | | | | | | |  |
| ICS= inhaled corticosteroids, Inh.=inhibitors, CVD=cardio-vascular disease | | | | | | | | | | | | | | | |  |  | |  |
| CVD medication includes: ACE inh., alpha/beta-blocker, Ca-channel-blocker, aspirin, anti-hypertension medication | | | | | | | | | | | | | | | |  |  | |  |
| Other: medication considered as not relevant for disease progression, such as anti-histamines, thyroid medication, or gastrointestinal medication | | | | | | | | | | | | | | | | | | | |
| Other comorbidities: comorbidities, that can not be derived from the medication list | | | | | | | | | | | | | | | | | | | |
| X=taken periodically within the 5 year follow up period | | | | | | | |  |  |  |  |  |  |  |  |  |  |  | |
| XX= taken since the year indicated and ongoing at visit 2012 | | | | | | | | |  |  |  |  |  |  |  |  |  |  | |
| year=year started with medication | | | | | | | | | | | | | | | | | | | |

**Table S2: List of all markers, which were shown to be repeatable in 2006/2007**

| **BAL** | **SPUTUM** |
| --- | --- |
| CD14+ monocytes | macrophages (%) |
| CD16+ neutrophils | neutrophils (%) |
|  | (non-squamous) epithelia cells (%) |
| total cell count |  |
| alpha1-antitrypsin | alpha1-antitrypsin |
| human serum albumin | human serum albumin |
| total-protein | interleukin 6 |
|  | matrix-metalloprotease 1 |
| calprotectin/ total-protein | matrix-metalloprotease 7 |
| interleukin 8/ total-protein | tissue inhibitor of metalloproteases 1 |
| neutrophil elastase/ total-protein |  |
| matrix-metalloprotease 9/total-protein | matrix-metalloprotease 9/total-protein |
| myeloperoxidase/total-protein |  |
|  |  |

| **SERUM** |  |
| --- | --- |
| calprotectin | interleukin 7 |
| creatinine | interleukin 8 |
| C-reactive protein | lipopolysaccharide binding protein |
| eotaxin | leptin |
| hepatocyte growth factor | CXCL9 |
| human serum albumin | CCL3 |
| interferon alpha | CCL4 |
| interferon gamma | matrix-metalloprotease 1 |
| insulin-like growthfactor-bindingprotein 1 | matrix-metalloprotease 9 |
| insulin-like growthfactor-bindingprotein 2 | neutrophil elastase |
| insulin-like growthfactor 1 | prostaglandin-derived growthfactor AA |
| insulin-like growthfactor 2 | prostaglandin-derived growthfactor AB/BB |
| interleukin 12p40/p70 | serotonin |
| interleukin 15 | transforming-growthfactor beta |
| interleukin 1B | tumor-nekrosisfactor alpha |
| interleukin 2 | vascular endothelia growthfactor |
| interleukin 2R | **URINE** |
| interleukin 6 | creatinine |

Blood cell markers not listed

**Table S3: Demographic data of an independent group of smoking COPD GOLD 2 patients from Grosshansdorf, Germany**

|  | 2006 | 2009 |
| --- | --- | --- |
|  | COPD smokers | COPD smokers |
|  | n=24 | n=24 |
| Female/male | 5/19 | 5/19 |
| Age [years]^a^ | 63 (56.66) | 66 (59.68) |
| BMI [kg/m2] | 28.9 ± 6.3 | 28.0 ± 6.2 |
| Pack-years | 56 ± 31 | 58 ± 31 |
| FEV1 [L] | 2.0 ± 0.4 | 1.7 ± 0.4 |
| FEV1 % pred. | 63.8 ± 8.2 | 57.0 ± 9.4 |
| FVC [L] | 3.6 ± 0.7 | 3.3 ± 0.6 |
| FEV1/FVC [%] | 55.5 ± 10.9 | 53.4 ± 11.2 |
| pO2 [mm Hg] | 72.6 ± 8.5 | 67.9 ± 9.5 |

^a^: presented as mean (minimum, maximum), all other data is presented as mean ± SD

**Table S4: Repeatability of blood cells and hematology markers between 2006 and 2009**

|  | r | ICC |
| --- | --- | --- |
|  |  |  |
| Leukocytes | 0.629 | 0.73 |
| Neutrophils (%) | 0.772 | 0.78 |
| Monocytes (%) | 0.576 | 0.71 |
| Thrombocytes | 0.709 | 0.72 |
| Erythrocytes% | 0.737 | 0.80 |
| Hemoglobin | 0.630 | 0.68 |
| Hematocrit | 0.498 | 0.61 |
| MCV | 0.795 | 0.82 |
| MCH | 0.761 | 0.77 |

MCH: mean corpuscular/cellular hemoglobin. MCV: mean corpuscular/cell volume
