# Supplementary figures and images for: Potential prognostic value of biomarkers in lavage, sputum and serum in a five year clinical follow-up of smokers with and without COPD
Source: BMC Pulm Med. 2014 Mar 1;14:30. doi: 10.1186/1471-2466-14-30 (PMC4021348; doi:10.1186/1471-2466-14-30)

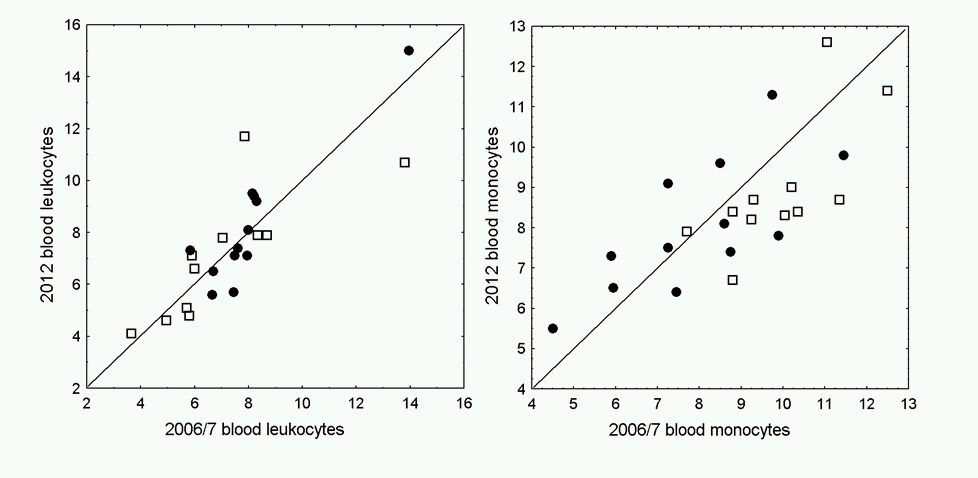

Supplement: Additional file 2: Figure S1 — Correlation for total blood leukocytes (left) and the percentage of monocytes (right) between 2006/2007 and 2012. Open symbols: smokers without COPD, closed symbols smokers with COPD. [file 1471-2466-14-30-S2.tif]
